# Synthesis, antitumour and antioxidant activities of novel α,β-unsaturated ketones and related heterocyclic analogues: EGFR inhibition and molecular modelling study

**DOI:** 10.1080/14756366.2018.1434519

**Published:** 2018-02-19

**Authors:** Walaa M. El-Husseiny, Magda A.-A. El-Sayed, Naglaa I. Abdel-Aziz, Adel S. El-Azab, Esam R. Ahmed, Alaa A.-M. Abdel-Aziz

**Affiliations:** a Department of Pharmaceutical Organic Chemistry, Faculty of Pharmacy, Mansoura University, Mansoura, Egypt;; b Department of Pharmaceutical Chemistry, Faculty of Pharmacy, Horus University, New Damietta, Egypt;; c Department of Medicinal Chemistry, Faculty of Pharmacy, Mansoura University, Mansoura, Egypt;; d Department of Pharmaceutical Chemistry, College of Pharmacy, King Saud University, Riyadh, Saudi Arabia;; e Department of Organic Chemistry, Faculty of Pharmacy, Al-Azhar University, Cairo, Egypt;; f Confirmatory Diagnostic Unit, Vacsera, Giza, Egypt

**Keywords:** α,β-Unsaturated ketone, antitumour activity, antioxidant effect, EGFR inhibition, molecular docking

## Abstract

New α,β-unsaturated ketones **4a**,**b**; **5a–c**; and **6a**,**b**; as well as 4-*H* pyran **7**; pyrazoline **8a**,**b**; isoxazoline **9**; pyridine **10–11**; and quinoline-4-carboxylic acid **12a**,**b** derivatives were synthesized and evaluated for *in vitro* antitumour activity against HepG2, MCF-7, HeLa, and PC-3 cancer cell lines. Antioxidant activity was investigated by the ability of these compounds to scavenge the 2,2′-azinobis(3-ethylbenzothiazoline-6-sulfonic acid) radical cation (ABTS^•+^). Compounds **6a**, **6b**, **7**, and **8b** exhibited potent antitumour activities against all tested cell lines with [IC_50_] ≅5.5–18.1 µΜ), in addition to significantly high ABTS^•+^ scavenging activities. *In vitro* EGFR kinase assay for **6a**, **6b**, **7**, and **8b** as the most potent antitumour compounds showed that; compounds **6b**, and **7** exhibited worthy EGFR inhibition activity with IC_50_ values of 0.56 and 1.6 µM, respectively, while compounds **6a** and **8b** showed good inhibition activity with IC_50_ values of 4.66 and 2.16 µM, respectively, compared with sorafenib reference drug (IC_50_ = 1.28 µM). Molecular modelling studies for compounds **6b**, **7**, and **8b** were conducted to exhibit the binding mode towards EGFR kinase, which showed similar interaction with erlotinib.

## Introduction

Cancer is a group of diseases involving abnormal cell growth with the potential to spread into or invade nearby tissues^1–3^. Although chemotherapy is the mainstay of cancer therapy, it produces substantial side effects that may be attributed to cytotoxic effects on normal cells^1–3^. This clearly underlies the urgent need for developing novel chemotherapeutic agents that will be more selective for cancer cells, and thus produce fewer side effects^1–12^. On the other hand, free radicals and the reactive oxygen species are constantly generated through many biological processes in the body^13^. The capability of antioxidants to reduce the risk of certain cancer types is linked to their ability to scavenge free radicals, reduce oxidative stress, and decrease abnormal cell division^13–17^. Administration of a single molecule acting through a different mechanism is a better drug candidate than drug combinations^18^. Hence, several studies have investigated both anticancer and antioxidant activities of numerous newly synthesized molecules^18–23^.

**Figure 1.**
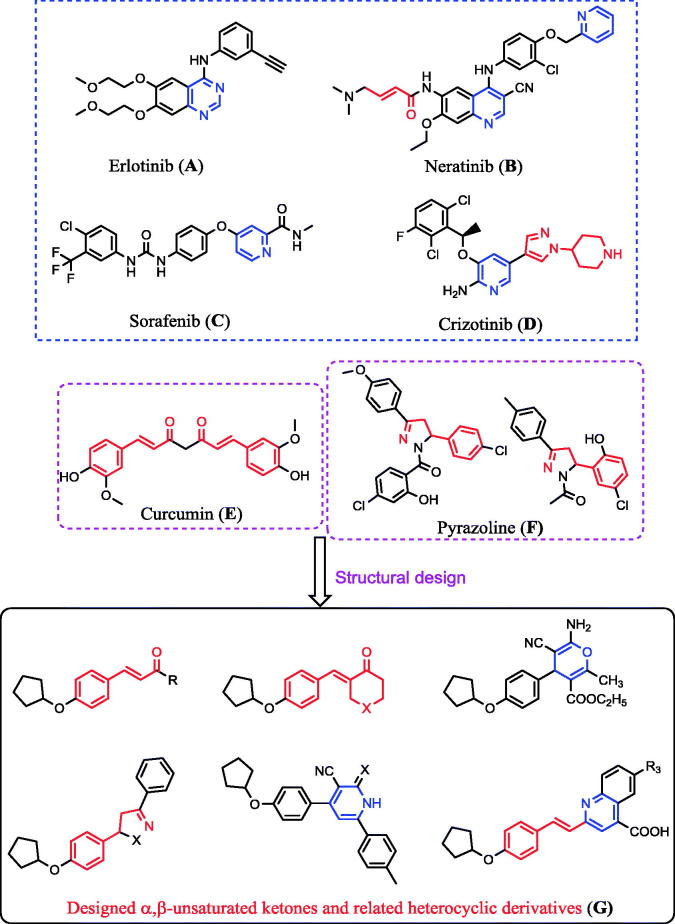
The reported antitumour (**A–F**) and the designed (**G**) compounds.

Furthermore, a high level of EGFR kinase enzyme is overexpressed in several tumours such as those in colon, prostate, breast, HeLa, HepG2, and non-small lung cancers^24–31^. The inhibition of EGFR kinase enzyme is used in cancer treatment, and is effected by blocking this enzyme with small molecules such as erlotinib (**A**)^32^^,^^33^, neratinib (**B**)^34–36^, sorafenib (**C**)^37^, and crizotinib (**D**)^38–40^ (Figure 1). Additionally, the α,β-unsaturated ketones, such as curcumin (**E**; Figure 1), are a major class of widespread natural products and constitute the core structure of many drugs covering a wide range of biological applications, including EGFR inhibition as well as antioxidant and antitumour activities^22^^,^^23^^,^^41–53^. Moreover, heterocycles such as pyrazoline (**F**; Figure 1)^54^^,^^55^, isoxazoline^56^, pyran^22^, pyridine^57^, and quinoline^58^ derivatives possess potent antioxidant and antitumour activities as well as some of these compounds possessed EGFR inhibition activities^53^^,^^59^^,^^60^.

Taking all the aforementioned facts into account in our continuous efforts to develop new structures to serve as antitumour and antioxidant agents, we synthesized new α,β-unsaturated ketones, 4-*H* pyran, pyrazoline, isoxazoline, pyridine, and quinoline derivatives (**G**; Figure 1). The rationale for evaluating the antitumour, antioxidant, and EGFR kinase inhibition activities of the designed molecules (**G**; Figure 1) was as follows: (i) design the structure–activity relationship for compounds incorporating α,β-unsaturated ketones with diverse substituent groups; (ii) recognise the effectiveness of the cyclic α,β-unsaturated ketones versus the acyclic derivatives; (iii) thus, compare the cycloalkanones and their piperidinone analogues; (iv) heterocyclic compounds resulting from the addition reaction of α,β-unsaturated ketones such as pyrane, pyrazoline, oxazoline, and pyridine derivatives were also included in the study in order to cover the most relative analogues.

Furthermore, the most active antitumour compounds were subjected to EGFR kinase inhibition test and docked into the binding sites of EGFR kinase enzyme to explore their complementarity with the specified binding pockets.

## Materials and methods

### Chemistry

Melting points (°C, uncorrected) were measured using a Fisher-Johns apparatus. Elemental analyses were carried out at the microanalytical unit, Cairo University. IR spectra (potassium bromide [KBr]) were acquired using a Mattson 5000 FT-IR spectrometer (ν in cm^−1^). ^1^H NMR and ^13^C NMR spectra were obtained in deuterated dimethyl sulphoxide (DMSO-d_6_) or deuterated chloroform (CDCl_3_) on Bruker 400 and 100 MHz instruments, respectively, using tetramethyl silane (TMS) as an internal standard. Chemical shifts were reported downfield from TMS in ppm, *δ* units. Mass spectrometry (MS) measurements were performed on a JEOL JMS-600H spectrometer. The purities of the compounds were evaluated by thin layer chromatography (TLC), which was performed on silica gel G (Merck), and spots were visualised by irradiation with ultraviolet light (UV; 254 nm). Compound **3**, 4-(cyclopentyloxy)benzaldehyde, was synthesized in accordance with the method described in the literature^61^.

### General method for the synthesis of α,β-unsaturated ketone derivatives (4a,b; 5a-c; and 6a,b)

A solution of 4-(cyclopentyloxy)benzaldehyde **3** (1.9 g, 0.01 mol) in ethanol (20 ml) was added to a stirred solution of the appropriate ketone (0.03 mol) in ethanol (20 ml) containing NaOH (0.8 g, 0.02 mol). The reaction mixture was refluxed for 8 h, cooled and the solvent was evaporated under reduced pressure. The resulting solid was triturated with diethyl ether, filtered, dried, and crystallised from the appropriate solvent.

#### 4-(4-(Cyclopentyloxy)phenyl)but-3-en-2-one (4a)

Crystallisation solvent, ethanol; Yield, 40%; melting point (mp): 219–220 °C; IR (KBr) *ν*_max_/cm^−1^ 1610 (C = O), 1530, 1525, 1510, 1470 (C = C). ^1^H NMR (DMSO-d_6_); *δ*: 7.95 (d, 2H, Ar-H, *J* = 8 Hz), 7.52 (d, 2H, Ar-H, *J* = 8 Hz), 7.28 (d, 1H, CH = CH, *J* = 8.4 Hz), 6.53 (d, 1H, CH = CH, *J* = 8.4 Hz), 4.80–4.70 (m, 1H, CH), 2.26 (s, 3H, CH_3_), 1.95–1.85 (m, 2H, CH_2_), 1.75–1.66 (m, 4H, 2CH_2_), 1.60–1.52 (m, 2H, CH_2_). MS *m*/*z* (%); 232.00 (6.39, M^+^+2), 231.00 (20.31, M^+^+1), 230.10 (21.46, M^+^), 224.00 (52.84), 147.00 (49.56), 142.10 (100.00), 121.00 (22.93), 100.00 (16.25). Anal. Calcd. for C_15_H_18_O_2_ (%): C, 78.23; H, 7.88. Found: C, 78.63; H, 8.18.

#### 3-(4-(Cyclopentyloxy)phenyl)-1-(4-methylphenyl)prop-2-en-1-one (4b)

Crystallisation solvent, water; Yield, 85%; mp: 220–222 °C; IR (KBr) *ν*_max_/cm^−1^ 1615 (C = O), 1540, 1535, 1525, 1480 (C = C). ^1^H NMR (CDCl_3_); *δ*: 7.85 (d, 4H, Ar-H, *J* = 8 Hz), 7.32 (d, 4H, Ar-H, *J* = 8 Hz), 7.18 (d, 1H, CH = CH, *J* = 8.4 Hz), 6.73 (d, 1H, CH = CH, *J* = 8.4 Hz), 4.80–4.72 (m, 1H, CH), 2.36 (s, 3H, CH_3_), 1.85–1.80 (m, 2H, CH_2_), 1.70–1.62 (m, 4H, 2CH_2_), 1.60–1.50 (m, 2H, CH_2_).^13^C NMR (DMSO-d_6_); *δ*: 198.2, 155.8, 143.3, 135.6, 134.3, 129.1, 128.4, 128.0, 114.8, 78.3, 32.2, 23.5, 21.0. MS *m*/*z* (%); 306.13 (17.66, M^+^), 238.10 (100.00), 237.08 (87.48), 210.08 (14.15), 209.07 (14.02), 195.06 (16.87), 144.03 (42.22). Anal. Calcd. for C_21_H_22_O_2_ (%): C, 82.32; H, 7.24. Found: C, 82.0 2; H, 7.05.

#### 2-(4-(Cyclopentyloxy)benzylidene)cyclopentanone (5a)

Crystallisation solvent, ethanol; Yield, 55%; mp: 282–283 °C; IR (KBr) *ν*_max_/cm^−1^ 1600 (C = O), 1535, 1520, 1510, 1500 (C = C). ^1^H NMR (DMSO-d_6_); *δ*: 7.55 (d, 2H, Ar-H, *J* = 8 Hz), 7.11 (brs, 1H, CH=), 6.90 (d, 2H, Ar-H, *J* = 8 Hz), 4.89–4.80 (m, 1H, CH), 3.20–3.10 (m, 2H, CH_2_), 2.11–1.88 (m, 4H, 2CH_2_), 1.72–1.61 (m, 4H, 2CH_2_), 1.60–1.45 (m, 4H, 2CH_2_). MS *m*/*z* (%); 257.08 (0.98, M^+^+1), 256.09 (1.28, M^+^), 146.05 (54.79), 145.04 (35.70), 131.03 (100.00), 117.06 (25.47), 115.03 (38.21), 107.02 (73.33). Anal. Calcd. for C_17_H_20_O_2_ (%): C, 79.65; H, 7.86. Found: C, 80.05; H, 8.06.

#### 2-(4-(Cyclopentyloxy)benzylidene)cyclohexanone (5b)

Crystallisation solvent, water; Yield, 50%; mp: 291–292 °C; IR (KBr) *ν*_max_/cm^−1^ 1620 (C = O), 1550, 1642, 1530, 1470 (C = C). ^1^H NMR (DMSO-d_6_); *δ*: 7.25 (d, 2H, Ar-H, *J* = 8 Hz), 7.00 (brs, 1H, CH=), 6.85 (d, 2H, Ar-H, *J* = 8 Hz), 4.90–4.82 (m, 1H, CH), 2.80 (t, 2H, CH_2_, *J* = 4.5 Hz), 2.40 (t, 2H, CH_2_, *J* = 4.5 Hz), 2.00–1.90 (m, 2H, CH_2_), 1.85–1.60 (m, 6H, 3CH_2_), 1.50–1.30 (m, 2H, CH_2_), 1.20–1.00 (m, 2H, CH_2_). MS *m*/*z* (%); 272.15 (2.78, M^+^+2), 271.13 (15.64, M^+^+1), 270.11 (52.26, M^+^), 203.08 (26.47), 202.07 (74.25), 201.07 (25.10), 145.05 (21.80), 107.02 (100.00). Anal. Calcd. for C_18_H_22_O_2_ (%): C, 79.96; H, 8.20. Found: C, 80.01; H, 8.50.

#### 2-(4-(Cyclopentyloxy)benzylidene)cycloheptanone (5c)

Crystallisation solvent, water; Yield, 60%; mp: 284–285 °C; IR (KBr) *ν*_max_/cm^−1^ 1625 (C = O), 1555, 1540, 1534, 1490 (C = C). ^1^H NMR (DMSO-d_6_); *δ*: 7.21 (d, 2H, Ar-H, *J* = 8.2 Hz), 7.02 (brs, 1H, CH=), 6.80 (d, 2H, Ar-H, *J* = 8.2 Hz), 4.60–4.50 (m, 1H, CH), 3.20–3.10 (m, 2H, CH_2_), 2.11–1.75 (m, 10H, 5CH_2_), 1.60–1.47 (m, 6H, 3CH_2_). MS *m*/*z* (%); 286.30 (0.2, M^+^+2), 285.20 (0.8, M^+^+1), 284.10 (35.00, M^+^), 216.10 (58.00), 215.10 (100.00), 121.10 (34.00), 120.10 (46.08), 41.10 (37.07). Anal. Calcd. for C_19_H_24_O_2_ (%): C, 80.24; H, 8.51. Found: C, 80.70; H, 8.91.

#### 3-(4-(Cyclopentyloxy)benzylidene)-1-methylpiperidin-4-one (6a)

Crystallisation solvent, water; Yield, 65%; mp: 248–250 °C. IR (KBr) *ν*_max_/cm^−1^ 1600 (C = O), 1540, 1525, 1535, 1490 (C = C). ^1^H NMR (, DMSO-d_6_); *δ*: 7.00 (d, 2H, Ar-H, *J* = 8 Hz), 6.80–6.71 (m, 3H, Ar-H, CH=), 4.60–4.50 (m, 1H, CH), 3.15 (s, 2H, CH_2_), 2.85 (t, 2H, CH_2,_*J* = 4.5 Hz), 2.60 (t, 2H, CH_2,_*J* = 4.5 Hz), 2.30 (s, 3H, N–CH_3_), 2.10–1.90 (m, 2H, CH_2_), 1.80–1.71 (m, 4H, 2CH_2_), 1.55–1.45 (m, 2H, CH_2_). MS *m*/*z* (%); 287.16 (6.34, M^+^+2), 286.15 (21.93, M^+^+1), 285.15 (8.16, M^+^), 166.05 (17.92), 161.06 (4.83), 112.08 (34.02), 111.09 (42.67), 110.02 (100.00). Anal. Calcd. for C_18_H_23_NO_2_ (%): C, 75.76; H, 8.12; N, 4.91. Found: C, 75.86; H, 8.22; N, 5.01.

#### 3-(4-(Cyclopentyloxy)benzylidene)-1-ethylpiperidin-4-one (6b)

Crystallisation solvent, water; Yield, 64%; mp: 240–241 °C. IR (KBr) *ν*_max_/cm^−1^ 1635 (C = O), 1560, 1550, 1530, 1485 (C = C). ^1^H NMR (DMSO-d_6_); *δ*: 7.20 (d, 2H, Ar-H, *J* = 8 Hz), 6.90–6.80 (m, 3H, Ar-H, CH=), 4.90–4.80 (m, 1H, CH), 3.70 (q, 2H, CH_2_CH_3,_*J* = 7 Hz), 3.20 (s, 2H, CH_2_), 2.85 (t, 2H, CH_2_, *J* = 4.5 Hz), 2.65 (m, 2H, CH_2_), 2.00–1.90 (m, 2H, CH_2_), 1.75–1.60 (m, 4H, 2CH_2_), 1.55–1.45 (m, 2H, CH_2_), 1.02 (t, 3H, CH_2_CH_3_,*J* = 7 Hz). MS *m*/*z* (%); 301.30 (18.00, M^+^+2), 300.20 (38.50, M^+^+1), 299.20 (26.00, M^+^), 137.90 (36.09), 132.90 (42.35), 123.90 (100.00), 72.10 (58.50), 58.00 (51.77). Anal. Calcd. for C_19_H_25_NO_2_ (%): C, 76.22; H, 8.42; N, 4.68. Found: C, 76.62; H, 8.72; N, 5.00.

### Synthesis of ethyl 6-amino-5-cyano-4-(4-(cyclopentyloxy)phenyl)-2-methyl-*4H*-pyran-3-carboxylate (7)

A mixture of 4-(cyclopentyloxy)benzaldehyde **3** (0.57 g, 0.003 mol), ethylacetoacetate (0.39 g, 0.003 mol), malononitrile (0.20 g, 0.003 mol), and sodium benzoate (15 mol%) in ethanol (20 ml) was stirred at room temperature for 24 h. The reaction mixture was filtered, and the solid product was washed with water and then with ethanol, dried and crystallised from dimethylformamide. Yield, 45%; mp >300 °C; IR (KBr) *ν*_max_/cm^−1^ 3401 and 3326 (NH_2_), 2221 (C≡N), 1697 (C = O). ^1^H NMR (DMSO-d_6_); *δ*: 8.30 (brs, 2H, NH_2_, D_2_O exchangeable), 7.30 (d, 2H, Ar-H, *J* = 8 Hz), 7.90 (d, 2H, Ar-H, *J* = 8 Hz), 5.70 (s,1H, 4-H of pyran), 5.10–5.00 (m, 1H, CH), 4.10 (q, 2H, CH_3_CH_2_O, *J* = 7.5 Hz), 2.50 (s, 3H, CH_3_), 2.00–1.90 (m, 2H, CH_2_), 1.80–1.60 (m, 6H, 3CH_2_), 1.20 (t, 3H, CH_3_CH_2_O, *J* = 7.5 Hz). MS *m*/*z* (%); 368.25 (0.81, M^+^), 348.07 (18.46), 321.06 (28.72), 276.07(91.20), 275.06 (68.87), 274.06 (28.04), 248.05 (17.64), 107.06 (100.00). Anal. Calcd. for C_21_H_24_N_2_O_4_ (%): C, 68.46; H, 6.57; N, 7.60. Found: C, 68.66; H, 7.00; N, 8.00.

### Synthesis of compounds 8a and 8b

A mixture of compound **4b** (0.91 g, 0.003 mol) and hydrazine hydrate (0.15 g, 0.003 mol) in absolute ethanol (30 ml) or phenyl hydrazine (0.32 g, 0.003 mol) in glacial acetic acid (5 ml) was heated under reflux for 9–10 h. After cooling, the separated products were filtered, dried, and crystallised from ethanol to yield the title compounds.

#### 5-(4-(Cyclopentyloxy)phenyl)-3-(4-methylphenyl)-4,5-dihydro-1H-pyrazole (8a)

Yield, 55%; mp: 145–146 °C. IR (KBr) *ν*_max_/cm^−1^ 3450 (NH), 1547 (C = N). ^1^H NMR (DMSO-d_6_); *δ*: 7.91–7.10 (m, 8H, Ar-H), 6.75 (dd, *J* = 11.7, 4.5 Hz, 1H, 5-H of pyrazoline), 4.92–4.82 (m, 1H, CH), 3.75 (dd, *J* = 11.7, 18.0 Hz, 1H, 4-H of pyrazoline), 3.50 (dd, *J* = 4.5, 18.0 Hz, 1H, 4-H of pyrazoline), 2.38 (s, 3H, CH_3_), 2.10–1.93 (m, 2H, CH_2_), 1.90–1.49 (m, 6H, 3CH_2_), 9.20 (brs, 1H, NH, D_2_O exchangeable). MS *m*/*z* (%); 321.30 (2.00, M^+^+1), 320.20 (6.50, M^+^), 318.90 (22.02), 261.00 (20.50), 145.10 (16.02), 143.90 (100.00), 120.10 (32.00), 90.90 (31.00). Anal. Calcd. for C_21_H_24_N_2_O (%): C, 78.71; H, 7.55; N, 8.74. Found: C, 79.01; H, 7.88; N, 8.95.

#### 5-(4-(Cyclopentyloxy)phenyl)-1-phenyl-3-(4-methylphenyl)-4,5-dihydro-1H-pyrazole (8b)

Yield, 60%; mp: 139–141 °C. IR (KBr) *ν*_max_/cm^−1^ 1547, 1560, 1550, 1545 (C = N, C = C). ^1^H NMR (DMSO-d_6_); *δ*: 7.00–7.90 (m, 13H, Ar-H), 6.85–6.75 (m, 1H, 5-H of pyrazoline), 4.80–4.70 (m, 1H, CH), 3.90–3.80 (m, 1H, 4-H of pyrazoline), 3.50–3.30 (m, 1H, 4-H of pyrazoline), 2.37 (s, 3H, CH_3_), 2.00–1.95 (m, 2H, CH_2_), 1.90–1.50 (m, 6H, 3CH_2_). MS *m*/*z* (%); 397.00 (10.81, M^+^+1), 396.40 (13.81, M^+^), 281.05 (36.86), 233.06 (26.59), 220.05 (100.00), 180.04 (36.11), 151.10 (25.03), 117.00 (53.87). Anal. Calcd. for C_27_H_28_N_2_O (%): C, C, 81.78; H, 7.12; N, 7.06. Found: C, 82.08; H, 7.22; N, 7.16.

### Synthesis of 5-(4-(cyclopentyloxy)phenyl)-3-(4-methylphenyl)-4,5-dihydroisoxazole (9)

A mixture of compound **4b** (0.91 g, 0.003 mol), hydroxylamine hydrochloride (0.2 g, 0.003 mol), and potassium hydroxide (0.2 g, 0.003 mol) in ethanol (20 ml) was refluxed for 12 h. The solvent was evaporated under reduced pressure and the residue obtained was triturated with water, filtered, and dried to yield compound **9**, which crystallised from ethanol. Yield, 56%; mp: 120–122 °C. IR (KBr) *ν*_max_/cm^−1^ 1540, 1565, 1555, 1545 (C = N, C = C). ^1^H NMR (DMSO-d_6_); *δ*: 7.90–7.30 (m, 8H, Ar-H), 6.80–6.70 (m,1H, 5-H of isoxazole), 4.80–4.70 (m, 1H, CH), 3.90–3.80 (m, 2H, 4-H of isoxazoline), 2.36 (s, 3H, CH_3_), 2.00–1.85 (m, 2H, CH_2_), 1.80–1.50 (m, 6H, 3CH_2_). MS *m*/*z* (%); 321.09 (0.81, M+), 292.05 (21.82), 291.05 (46.86), 236.06 (36.59), 222.05 (100.00), 179.04 (26.11), 161.10 (25.93), 118.00 (63.87). Anal. Calcd. for C_21_H_23_NO_2_ (%): C, 78.47; H, 7.21; N, 4.36. Found: C, 78.90; H, 7.60; N, 4.96.

### Synthesis of 4-(4-(cyclopentyloxy)phenyl)-2-oxo-6-(4-methylphenyl)-1,2-dihydropyridine-3-carbonitrile (10)

A mixture of compound **4b** (1.53 g, 0.005 mol), ethylcyanoacetate (0.56 g, 0.005 mol), and ammonium acetate (3.1 g, 0.04 mol) in absolute ethanol (50 ml) was refluxed for 8 h. After cooling, the product was collected by filtration, washed with ethanol, dried, and crystallised from ethanol to yield the title compound. Yield, 40%; mp: 278–280 °C; IR (KBr) *ν*_max_/cm^−1^ 3445 (NH), 2215 (C≡N), 1652 (C = O). ^1^H NMR (DMSO-d_6_); *δ*: 8.28–8.04 (m, 3H, Ar-H), 8.07 (br, s, 1H, NH, D_2_O exchangeable), 8.10–7.92 (m, 2H, Ar-H), 7.40–7.28 (m, 2H, Ar-H), 7.10–7.05 (m, 2H, Ar-H), 5.00–4.95 (m, 1H, CH), 2.39 (s, 3H, CH_3_), 2.10–1.95 (m, 2H, CH_2_), 1.85–1.61 (m, 6H, 3CH_2_). MS *m*/*z* (%); 370.00 (15.81, M^+^), 255.05 (21.12), 285.05 (38.06), 230.06 (29.49), 229.05 (100.00), 188.04 (66.11), 153.10 (15.13), 119.00 (50.7 7). Anal. Calcd. for C_24_H_22_N_2_O_2_ (%): C, 77.81; H, 5.99; N, 7.56. Found: C, 78.01; H, 6.10; N, 7.99.

### Synthesis of 2-amino-4-(4-(cyclopentyloxy)phenyl)-6-(4-methylphenyl)nicotinonitrile (11)

A mixture of compound **4b** (1.53 g, 0.005 mol), malononitrile (0.30 g, 0.005 mol), and ammonium acetate (3.1 g, 0.04 mol) in absolute ethanol (50 ml) was refluxed for 10 h. The reaction mixture was then cooled, poured into crushed ice, and the product separated out was filtered, washed with water, dried, and crystallised from water to yield compound **11**. Yield, 45%; mp: 272–273 °C; IR (KBr) *ν*_max_/cm^−1^ 3406 and 3336 (NH_2_), 2212 (C≡N), 1599(C = N). ^1^H NMR (DMSO-d_6_); *δ*: 8.29–8.19 (m, 3H, Ar-H), 8.10 (brs, 2H, NH_2_, D_2_O exchangeable), 7.90 (d, 2H, Ar-H, *J* = 4 Hz), 7.40 (d, 2H, Ar-H, *J* = 4 Hz), 7.10 (d, 2H, Ar-H, *J* = 4 Hz), 5.00–4.90 (m, 1H, CH), 2.40 (s, 3H, CH_3_), 2.10–2.00 (m, 2H, CH_2_), 1.90–1.75 (m, 4H, 2CH_2_), 1.70–1.60 (m, 2H, CH_2_). MS *m*/*z* (%); 369.14 (1.17, M^+^), 334.10 (20.39), 307.08 (19.44), 241.05 (20.53), 182.92 (13.80), 160.09 (35.90), 146.25 (100.00), 107.07 (24.46). Anal. Calcd. for C_24_H_23_N_3_O (%): C, 78.02; H, 6.27; N, 11.37. Found: C, 78.42; H, 6.66; N, 11.55.

### General method for the synthesis of 2-(4-(cyclopentyloxy)styryl)-6-substituted quinoline-4-carboxylic acids (12a,b)

A mixture of compound **4a** (1.15 g, 0.005 mol) and isatin derivatives (0.005 mol) in 50% aqueous ethanol (40 ml) containing potassium hydroxide (1.28 g, 0.023 mol) was refluxed for 24 h. The reaction mixture was filtered, and the filtrate was acidified with acetic acid and the solvent was evaporated under reduced pressure. The residue obtained was triturated with water, filtered, and dried to yield compounds **12a**,**b** which crystallised from dimethylformamide.

#### 6-Bromo-2-(4-(cyclopentyloxy)styryl)quinoline-4-carboxylicacid (12a)

Yield, 40%; mp >300 °C; IR (KBr) *ν*_max_/cm^−1^ 3425 (OH), 1640 (C = O), 1565 (C = N). ^1^H NMR (CDCl_3_); *δ* 11.30 (brs, 1H, OH, D_2_O exchangeable), 7.78–6.88 (m, 10H, CH = CH, Ar-H), 4.80–4.70 (m, 1H, CH), 2.00–1.90 (m, 2H, CH_2_), 1.70–1.60 (m, 4H, 2CH_2_), 1.50–1.40 (m, 2H, CH_2_). MS *m*/*z* (%); 439.00 (12.41, M^+^+2), 438.00 (16.05, M^+^+1), 437.00 (13.00, M^+^), 239.20 (56.20), 145.10 (32.00), 97.10 (57.01), 94.90 (71.00), 71.10 (100.00), Anal. Calcd. for C_23_H_20_BrNO_3_ (%): C, 63.02; H, 4.60; Br, 18.23; N, 3.20. Found: C, 63.42; H, 4.70; Br, 18.00; N, 2.92.

#### 2-(4-(Cyclopentyloxy)styryl)-6-fluoroquinoline-4-carboxylicacid (12b)

Yield, 50%; mp >300 °C; IR (KBr) *ν*_max_/cm^−1^ 3421 (OH), 1690 (C = O), 1577 (-C = N-). ^1^H NMR (CDCl_3_); *δ* 11.40 (brs, 1H, OH, D_2_O exchangeable), 7.88–6.42 (m, 10H, CH = CH, Ar-H), 4.90–4.82 (m, 1H, CH), 2.10–1.90 (m, 2H, CH_2_), 1.80–1.70 (m, 4H, 2CH_2_), 1.55–1.45 (m, 2H, CH_2_). ^13^C NMR (DMSO-d_6_); *δ*: 183.2, 158.8, 149.6, 140.0, 135.1, 134.2, 128.1, 126.8, 144.3, 144.3, 82.1, 32.2, 24.1. MS *m*/*z* (%); 377.07 (0.86, M^+^), 373.99 (40.47), 283.00 (23.66), 270.01 (15.00), 240.07 (10.76), 187.07 (25.35), 151.04 (11.07), 142.12 (100.00). Anal. Calcd. for C_23_H_20_FNO_3_ (%): C, 73.20; H, 5.34; F, 5.03; N, 3.71. Found: C, 73.30; H, 5.34; F, 5.33; N, 4.01.

### Biological testing

#### Antitumour evaluation

The evaluation of the antitumour activity was performed using tetrazolium salt MTT (3-(4,5-dimethyl-2-thiazolyl)-2,5-diphenyl-2*H*-tetrazolium bromide) assay as reported^62^^,^^63^.

#### Antioxidant assay

The absorbance (*A*_control_) of a green-blue solution (ABTS^+^ radical solution) resulted from a mixture of ABTS and manganese dioxide (MnO_2_) and was recorded at *λ*_max_ 734 nm, according to the reported procedure^64^^,^^65^. The absorbance (*A*_test_) was measured upon the addition of 20 µl of 1 mg/ml solution of the test sample in spectroscopic grade methanol/phosphate buffer (1:1 v/v) to the ABTS solution. The decrease in absorbance is expressed as % inhibition, which can be calculated from the following equation:

$$\%\text{ inhibition}\text{ = }\frac{A_{\text{control}}\text{-}A_{\text{test}}}{A_{\text{control}}}\times \text{100}\text{.}$$

L-Ascorbic acid 20 µl (2 mM) solution was used as standard antioxidant (positive control). A blank sample was run using only methanol/phosphate buffer (1:1), while the negative control was run with ABTS and the methanol/phosphate buffer.

#### EGFR kinase inhibition assay

EGFR kinase activity was determined via EGFR Human In-Cell ELISA Kit in 96-well plates according to the manufacturer's instructions (EGFR Kinase Assay Kit Catalog # ab126419 of ABCAM, Cambridge, MA), as supplemental information^66^. The EGFR kinase activities for each compound were expressed as IC_50_ values using seven concentrations (10.0, 5.0, 2.5, 1.25, 0.625, 0.31, and 0.15 µM).

### Docking methodology

All modelling experiments were conducted with MOE programs running on PC computer (MOE 2008.10 of Chemical Computing Group. Inc, Montreal, QC, Canada)^67^. Starting coordinates of the X-ray crystal structure of EGFR enzyme in complex with eroltinib (PDB code 1M17) is obtained from the RCSB Protein Data Bank. All the hydrogen was added and enzyme structure was subjected to a refinement. The docking methodology was similar to that described in our previous reports^5^^,^^68–70^.

## Results and discussion

### Chemistry

#### Synthesis of compounds 4–7 (Scheme 1)

The compound 4-(cyclopentyloxy)benzaldehyde (**3**) was obtained as a key intermediate in a 75% yield by the reaction of 4-hydroxybenzaldehyde (**1**) with bromocyclopentane (**2**) in the presence of phase-transfer catalyst; *t*-butylammonium bromide (Bu_4_NBr). Condensation of 4-(cyclopentyloxy)benzaldehyde (**3**) with various aliphatic, aromatic, cyclic, and heterocyclic ketones in an ethanolic solution of sodium hydroxide afforded the corresponding compounds **4a**,**b**; **5a–c**; and **6a**,**b**. The structures of the synthesized compounds were confirmed by their elemental and spectral analyses. Proton nuclear magnetic resonance (^1^H NMR) spectra of compounds **4a** and **4b** were confirmed by two doublets of vinylic protons at 7.28, 6.53, and 7.18, 6.73 ppm, respectively.

**Scheme 1.**
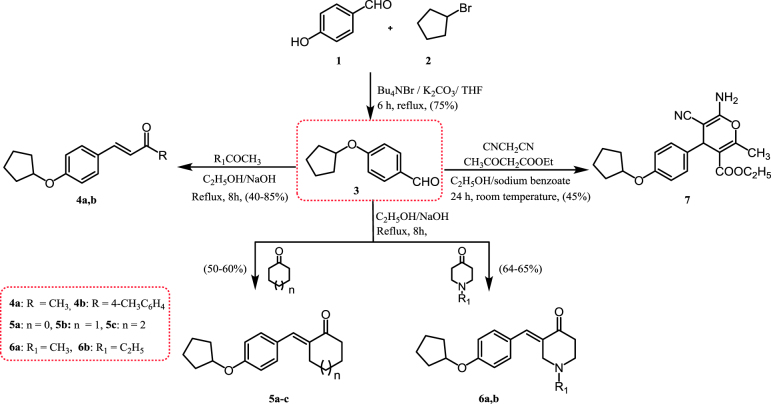
Synthesis of the designed α,β-unsaturated ketones and 4-*H* pyran derivatives.

Moreover, the ^1^H NMR spectrum of compound **4a** showed a singlet signal at 2.26 ppm attributed to an acetyl group. The ^1^H NMR spectrum compound **4b** was verified by the presence of new aromatic signals at 7.85–7.32 ppm in addition to a singlet signal at 2.36 ppm due to the presence of a 4-methyl group. The presence of a new peak at 198.2 ppm due to a carbonyl (CO) group was demonstrated in ^13^C NMR spectrum.

^1^H NMR spectra of compounds **5a–c** were characterised by the presence of cycloalkane protons at 4.90–1.00 ppm. The ^1^H NMR spectrum of compound **6a** is characterised by the presence of a singlet peak at 2.3 ppm corresponding to the methyl protons of the *N*-CH_3_ group, while a triplet–quartet pattern characteristic of an ethyl group (*N*-CH_2_CH_3_) was identified in the ^1^H NMR spectrum of compound **6b** at 2.65 and 3.70 ppm, respectively. Synthesis of 4-*H* pyran derivative (**7**) was achieved by stirring 4-(cyclopentyloxy)benzaldehyde (**3**), malononitrile, and ethyl acetoacetate in ethanol in the presence of a catalytic amount of sodium benzoate at room temperature. The infra-red (IR) spectrum of compound **7** exhibited bands at 3401, 3326 (NH_2_), 2221 (C≡N), and 1697 (C = O) cm^−1^_._ Meanwhile, the ^1^H NMR spectrum showed a triplet and quartet at 1.20 and 4.10 ppm integrating for the COOCH_2_CH_3_ group, respectively. In addition, presence of two singlet peaks at 5.70 and 8.30 ppm for the methyl (CH_3_) and amine (NH_2_) groups, respectively.

#### Synthesis of compounds 8–12 (Scheme 2)

The compound 3-(4-(cyclopentyloxy)phenyl)-1-(4-methylphenyl)prop-2-en-1-one (**4b**) was heated under reflux with hydrazine hydrate or phenylhydrazine in ethanol or glacial acetic acid, resulting in the corresponding pyrazoline derivatives **8a** and **8b**. ^1^H NMR spectra of compounds **8a** and **8b** were characterised by the disappearance of the olefinic protons with the appearance of pyrazoline protons at 6.85–6.75, 3.90–3.75, and 3.50–3.30 ppm. Moreover, facile cyclocondensation of compound **4b** with hydroxylamine hydrochloride in ethanolic potassium hydroxide gave the corresponding isoxazoline (**9**). The ^1^H NMR spectrum of compound **9** was characterised by the disappearance of the olefinic protons with the appearance of isoxazoline protons at 6.80–6.70 and 3.90–3.80 ppm. Reaction of the α,β-unsaturated ketone **4b** with ethylcyanoacetate or malononitrile in ethanol in the presence of ammonium acetate yielded the cyanopyridine derivatives **10** and **11**, respectively. IR spectra of compounds **10** and **11** were used to verify their structures through the appearance of characteristic absorption bands due to nitrile groups at 2215 and 2212 cm^−1^, respectively. In addition, a singlet peak at 8.07 ppm corresponding to the NH proton appeared in the ^1^H NMR spectrum of compound **10**, while a singlet peak at 8.10 ppm was assignable to the NH_2_ group in compound **11**, and both were deuterium oxide (D_2_O) exchangeable. Quinoline-4-carboxylic acid derivatives **12a**,**b** were prepared by condensation of 4-(4-(cyclopentyloxy)phenyl)but-3-en-2-one (**4a**) and isatin derivatives in ethanolic potassium hydroxide^71^. The IR spectrum of compound **12b** was characterised by the presence of absorption bands at 3421 cm^−1^ and 1690 cm^−1^, representing hydroxy (OH) and carbonyl (C = O) groups, respectively. Moreover, a broad singlet at 11.40 ppm assignable to the exchangeable OH group was seen in the ^1^H NMR spectrum, and the ^13^C NMR spectrum showed the presence of a signal for the carbonyl group at 183.20 ppm.

**Scheme 2.**
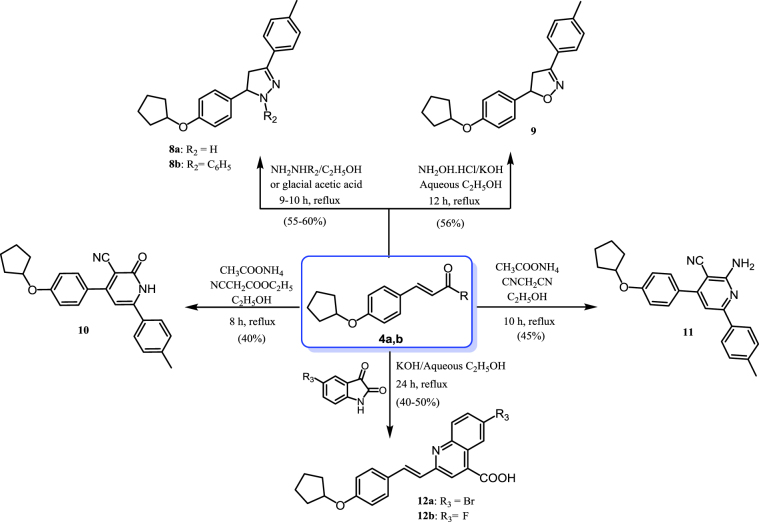
Synthesis of the designed pyrazoline, isoxazoline, cyanopyridine, and quinoline-4-carboxylic acid derivatives.

### Biological evaluation

#### Antitumour evaluation using MTT assay

The designed compounds were evaluated for their *in vitro* antitumour effects via the standard 3-(4,5-dimethylthiazol-2-yl)-2,5-diphenyltetrazolium bromide (MTT) method against a panel of four human tumour cell lines; namely, hepatocellular carcinoma cell line (HepG2), breast cancer cell line (MCF-7), human cervical cancer cell line (HeLa), and prostate cancer cell line (PC-3)^62^^,^^63^^,^^72^. The antitumour activities of the designed compounds **4–12**, along with that of the reference drugs 5-FU and afatinib are shown in Table 1. The α,β-unsaturated ketone **4a** showed moderate antitumour activity against the investigated cell lines (IC_50_ ≅ 21.8–40.9 µM), while replacement of the methyl moiety in α,β-unsaturated ketone **4a** with the 4-tolyl fragment in α,β-unsaturated ketone **4b** resulted in slightly increase in antitumour activity against HepG2, MCF-7, HeLa, and PC-3 cell lines, with IC_50_ values at 20.0, 36.4, 18.8, and 17.1 µM, respectively. Weak antitumour activity was demonstrated by the 2-arylidene cyclic ketones **5a–c** as shown by their IC_50_ values (30.1 to >100 µM). Interestingly, the 3-arylidene derivatives of piperidone **6a** and **6b** exhibited the greatest antitumour activities among the designed α,β-unsaturated ketone derivatives. For example, compound **6b** displayed very strong antitumour effects against HeLa and PC-3 cell lines, as expressed by IC_50_ values of 6.7 and 9.1 µM, respectively. Moreover, compound **6b** exhibited a strong inhibitory effect on the growth of HepG2 and MCF-7 cell lines, with IC_50_ values at 13.0 and 13.7 µM, respectively.

**Table 1.** *In vitro* antitumour activity of 5-fluorouracil, afatinib, and the tested compounds.

	IC_50_ (μM)^a^			
Compd no.	HepG2^b^	MCF-7^c^	HeLa^d^	PC-3^e^
**5-FU**	7.9 ± 0.17	5.4 ± 0.20	4.8 ± 0.21	8.3 ± 0.35
**Afatinib**	5.4 ± 0.25	7.1 ± 0.49	6.2 ± 0.67	7.7 ± 0.57
**4a**	27.3 ± 1.96	40.9 ± 2.79	25.7 ± 1.97	21.8 ± 1.68
**4b**	20.0 ± 1.11	36.4 ± 2.60	18.8 ± 1.57	17.1 ± 1.58
**5a**	>100	>100	77.8 ± 4.41	94.1 ± 5.82
**5b**	55.4 ± 3.95	49.4 ± 3.16	30.1 ± 2.24	71.1 ± 4.93
**5c**	71.3 ± 4.53	64.7 ± 4.27	37.5 ± 2.81	26.9 ± 1.89
**6a**	15.9 ± 1.02	18.1 ± 1.58	9.4 ± 0.98	10.5 ± 0.97
**6b**	13.0 ± 0.87	13.7 ± 1.35	6.7 ± 0.67	9.1 ± 0.88
**7**	8.0 ± 0.38	7.5 ± 0.54	10.3 ± 1.13	13.3 ± 1.26
**8a**	18.9 ± 1.35	29.3 ± 1.97	16.2 ± 1.36	12.7 ± 1.13
**8b**	7.2 ± 0.24	5.6 ± 0.36	5.5 ± 0.45	7.8 ± 0.56
**9**	62.3 ± 4.10	58.4 ± 4.50	46.2 ± 3.30	50.1 ± 3.55
**10**	80.9 ± 5.34	70.9 ± 4.98	51.2 ± 3.82	41.9 ± 2.87
**11**	92.9 ± 5.82	97.3 ± 5.51	62.4 ± 3.80	87.7 ± 5.41
**12a**	85.4 ± 5.31	87.1 ± 5.24	89.4 ± 4.89	>100
**12b**	30.8 ± 2.07	48.1 ± 3.25	66.8 ± 4.07	69.4 ± 4.32

a IC_50_, compound concentration required to inhibit tumour cell proliferation by 50% (mean ± SD, *n* = 3).

b Human hepato-cellular carcinoma cell line (HepG2).

c Human breast adenocarcinoma cell line (MCF-7).

d Human cervical epithelioid carcinoma cell line (HeLa).

e Human prostate cancer cell line (PC-3).

IC_50_, (μM): 1–10 (very strong), 11–25 (strong), 26–50 (moderate), 51–100 (weak), above 100 (non-cytotoxic).

More interestingly, compound **7**, which contained a 4-*H* pyran core, exerted good activities against HepG2 (IC_50_ = 8.0 µM), MCF-7 (IC_50_ = 7.5 µM), HeLa (IC_50_ = 10.3 µM), and PC-3 (IC_50_ = 13.3 µM) cancer cell lines. Moreover, *N*-phenylpyrazoline **8b** showed a sharp increase in antitumour activity when compared with α,β-unsaturated ketone analogue **4b**. IC_50_ values of compound **8b** against HepG2, MCF-7, HeLa, and PC-3 cell lines were 7.2, 5.6, 5.5, and 7.8 µM, respectively, in comparison with IC_50_ values of the reference drugs 5-FU (7.9, 5.4, 4.8, and 8.3 µM, respectively) and afatinib (5.4, 7.1, 6.2, and 7.6 µM, respectively). In addition, replacement of the phenyl ring in compound **8b** with the hydrogen atom in pyrazoline **8a** led to a decrease in antitumour activity against the MCF-7 cell line (IC_50_ = 29.3 µM), HepG2 (IC_50_ = 18.9 µM), HeLa (IC_50_ = 16.2 µM), and PC-3 (IC_50_ = 12.7 µM) cell lines. However, cyclisation of compounds **4a**,**b** to isoxazoline **9**; pyridines **10**–**11**; and quinolines **12a**,**b** analogues produced moderate to weak antitumour activity with IC_50_ values in the range of 30.8–97.3 µM.

#### *Antioxidant activity using* ABTS^•+^*radical-scavenging assay*

The assay is based on measuring the ability of the tested compounds to scavenge the long-life radical cation of ABTS^22^^,^^43^^,^^64^^,^^65^. In this study, all the newly synthesized compounds **4–12** and L-ascorbic acid, as a positive control, were evaluated and showed considerable free radical-scavenging activities. The reduction in colour intensity was expressed as inhibition percentage of the ABTS**^•^**^+^ as shown in Table 2. From the listed results, we concluded that all tested compounds exhibited more than 50% inhibition of the ABTS radical cation except derivatives **5a**, **10**, **11**, and **12a**. It is clear that the conversion of α,β-unsaturated ketones **4a**,**b** (% inhibition =52%) to the corresponding heterocyclic molecules generally led to sharp increases in antioxidant effects. Among them, the *N*-phenylpyrazoline derivative **8b** displayed the highest free radical-trapping properties, with 88.5% inhibition, which was comparable to L-ascorbic acid at 90.0%. Moreover, 4-*H* pyran **7** and isoxazoline **9** derivatives showed inhibition of 75.8% and 60.0%, respectively. Conversion of the acyclic α,β-unsaturated ketones **4a**,**b** (52% inhibition) into their corresponding cyclic α,β-unsaturated ketones **5a–c** (51% inhibition) and **6a**,**b** (54–55% inhibition) showed no change in activity. However, we concluded that compounds characterised by having pyrane **7**, pyrazoline **8b**, and isoxazoline **9** ring systems were among the most active compounds (60–88.5% inhibition), indicating that these core structures may play a role in trapping ABTS free radicals.

**Table 2.** The percentage inhibition of the ABTS radical cation by L-ascorbic acid and the tested compounds.

Compound no	Absorbance	%Inhibition
Control of ABTS	0.512	0
Ascorbic acid	0.051	90.0
**4a**	0.245	52.0
**4b**	0.243	52.6
**5a**	0.281	45.0
**5b**	0.249	51.4
**5c**	0.251	51.0
**6a**	0.234	54.3
**6b**	0.229	55.1
**7**	0.124	75.8
**8a**	0.240	53.0
**8b**	0.058	88.5
**9**	0.204	60.0
**10**	0.270	47.3
**11**	0.279	45.5
**12a**	0.275	46.3
**12b**	0.256	50.0

#### Correlations between antioxidant and antitumour activities

**Figure 2.**
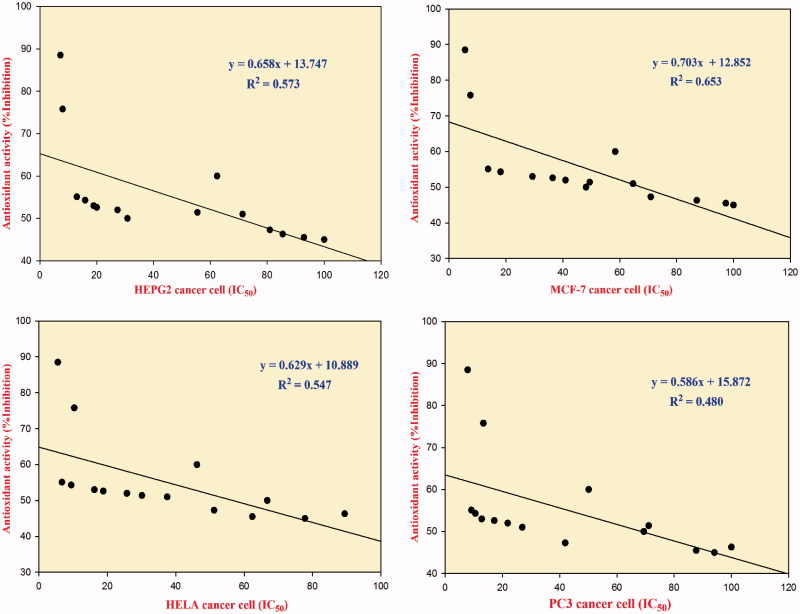
The overall correlation between the antioxidant activity (%Inhibition) and the antitumour activity of the synthesized compounds against cancer cell lines (HepG2, MCF-7, HeLa, and PC-3 cells).

The correlation between the antioxidant and the antitumour activities was investigated using SigmaPlot software (London, UK)^73^. The overall correlation between the antioxidant and antitumour activities of the synthesized compounds against individual cancer cell lines is shown in Figure 2. Most of the synthesized compounds showed moderate correlation (a moderate uphill relationship) between antioxidant and antitumour activities, as indicated by their coefficients of determination (*R*^2^). These *R*^2^ values were 0.573 (HepG2 cancer cell), 0.653 (MCF-7 cancer cell), 0.547 (HeLa cancer cell), and 0.480 (PC-3 cancer cell). The results indicate only a moderate linear relationship between the antioxidant and antitumour activities, which lead to the conclusion that antioxidant activity is not the only mechanism responsible for antitumour activity.

#### EGFR inhibitory activity

The antitumour activity results of compounds **6a**, **6b**, **7**, and **8b** encourage us to study the mechanism of antitumour activity using ELISA-based EGFR-TK assay with sorafenib as the reference drug^66^. The % inhibition and IC_50_ values of the tested compounds were calculated and are listed in Table 3. Compound **6b** and **7** revealed worthy EGFR inhibition activity with IC_50_ value of 0.56 and 1.6 µM, respectively, while compound **8b** showed good inhibitory activity against EGFR with IC_50_ value of 2.16 µM, compared to sorafenib reference drug (IC_50_ = 1.28 µM). On the other hand, compounds **6a** showed moderate inhibitory activity against EGFR with IC_50_ value of 4.66 µM, comparable to those of sorafenib (IC_50_ = 1.28 µM). We concluded, based on these results, that the designed compounds such as **6a**, **6b**, **7**, and **8b** are EGFR inhibitors which could be a new scaffold for the design of future analogues.

**Table 3.** *In vitro* IC_50_ values of the designed compounds towards EGFR kinase enzyme.

	% Inhibition							
Compd no.	10.0^a^	5.0^a^	2.5^a^	1.25^a^	0.625^a^	0.31^a^	0.15^a^	EGFR IC_50_ (μM)
**6a**	57.65	50.34	44.35	34.55	26.84	18.16	6.67	4.66
**6b**	83.66	76.71	64.44	61.23	55.33	48.95	26.52	0.56
**7**	72.78	65.98	52.86	49.65	43.27	31.78	8.22	1.6
**8b**	64.88	60.72	50.51	47.28	43.85	26.25	6.96	2.16
**Sorafenib**	80.88	71.63	56.72	49.48	43.27	33.92	10.82	1.28

a Concentarion in μM.

### Molecular docking results

The preceding results encouraged us to study the molecular docking of the most active compounds **6b**, **7**, and **8b** using EGFR, which are overexpressed in numerous tumours such as prostate (PC-3), breast (MCF-7), hepatocellular carcinoma (HepG2), and human cervical (HeLa) cancer cell lines^24–32^. All docking calculations were performed using MOE 2008.10 software^67^.

**Figure 3.**
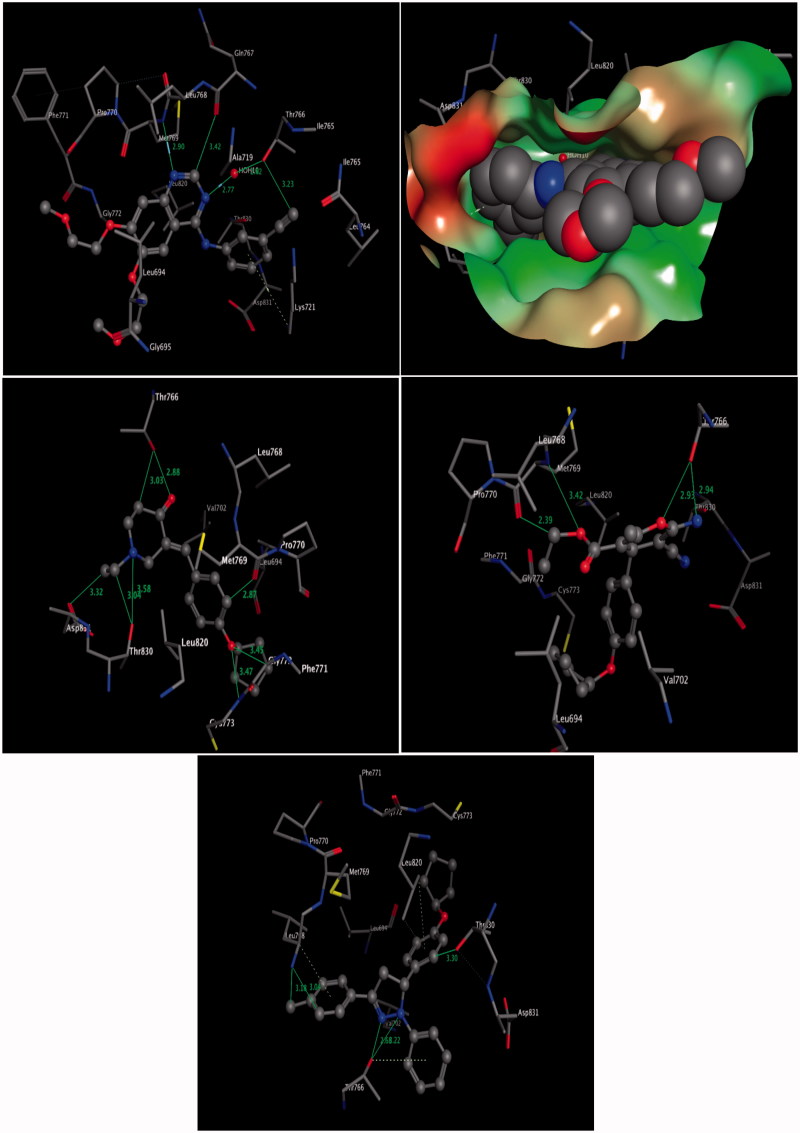
Three-dimensional (3D) interactions of erlotinib (upper panel), compounds **6b** (middle left panel), **7** (middle right panel), and **8b** (lower panel) with the receptor pocket of EGFR kinase. Hydrogen bonds are shown as green lines and CH–π interactions as dotted lines.

The docked compounds **6b**, **7**, **8b**, and the reference inhibitor erlotinib (Protein Data Bank [PDB] code 1M17)^33^ into the putative active site of EGFR are shown in Figure 3. The molecular modelling results of the compound, **6b**, demonstrated an approximate orientation of the molecule in comparison with erlotinib inside the putative binding site of receptor pocket with some additional hydrogen bond interactions with surrounding amino acids. These docking results showed three classical and five non-classical hydrogen bonds, where the distinctive residue Thr^766^ formed bifurcated hydrogen bonds with oxygen and carbon atoms of the piperidin-4-one ring system (Figure 3, middle left panel). In addition, the amino acid residue Thr^830^ formed bifurcated hydrogen bonds through NH–aliphatic-CH and NH–N interactions of *N*-ethylpiperidin-4-one, while the amino acid Asp^831^ showed another hydrogen bond with *N*-ethyl group of piperidin-4-one core through the C = O–aliphatic–CH interaction. Additionally, the surrounding amino acids Met^769^, and Gly^772^ showed another three interactions with aromatic ring and pentyloxy moiety through C = O–Aromatic-CH, O–aliphatic-CH and O–NH bonds (Figure 3, middle left panel).

Similarly, compound **7** binds into the putative active site of EGFR with three classical and one non-classical hydrogen bond. It was found that the amino acid Thr^766^ formed bifurcated classical hydrogen bonds with the 2-amino moiety and the oxygen atom of the 4-*H* pyran ring system (Figure 3, middle right panel). Moreover, the distinctive amino acid residue Met^769^ was involved in two hydrogen bonds: with the oxygen atom and with alkyl moieties of the ester group.

Moreover, compound, **8b**, demonstrated similar results as compounds **6b** and **7** inside the putative binding site of receptor pocket. These docking results showed two classical hydrogen bonds, where the distinctive residue Thr^766^ formed bifurcated classical hydrogen bonds with nitrogen atoms of the pyrazoline ring system (Figure 3, lower panel). In addition, three non-classical hydrogen bonds formed with surrounding amino acids, as shown in Figure 3 (lower panel). The amino acid residue Leu^768^ formed bifurcated hydrogen bonds through NH—Ar-CH interaction and one with the methyl group of the 4-tolyl moiety (NH—aliphatic-CH), while the third non-classical hydrogen bond was observed between the amino acid Thr^830^ and an aromatic ring through the OH—Ar-CH interaction. Additionally, the surrounding amino acids Leu^768^, Leu^820^, and Thr^766^ showed hydrophobic interactions with aromatic rings through CH—π and OH—π (Figure 3, lower panel).

## Conclusions

Novel α,β-unsaturated ketone **4**–**6a**,**b**, 4-*H* pyran **7**, pyrazoline **8a**,**b**, isoxazoline **9**, pyridine **10**–**11**, and quinoline-4-carboxylic acid **12a**,**b** derivatives have been synthesized, and the antitumour, antioxidant, and EGFR kinase inhibition activities have been evaluated. It is clear that most of the synthesized compounds exert significant antitumour activities. Among the tested derivatives, **6a**, **6b**, **7**, and **8b** showed potent IC_50_ values ≅ 5.5–18.1 µM, which were comparable to that of 5-FU (IC_50_ ≅ 4.8–8.3 µM) and afatinib (IC_50_ ≅ 5.4–7.6 µM). Moreover, compound **8b** has been shown promising, broad spectrum antitumour activity against the tested cell lines with an IC_50_ range of 5.5–7.8 µM. Additionally, compounds **6a**, **6b**, **7**, **8b**, and **9** exhibited the highest antioxidant effects using the ABTS radical-scavenging assay. Moreover, we observed a moderate relationship between the antitumour activity and the antioxidant effects of the tested compounds, which suggested that antioxidant effect is not the major role in the antitumour activity. Additionally, compounds **6b**, and **7** exhibited excellent inhibition towards EGFR kinase enzyme with IC_50_ values range of 0.56–1.6 µM, respectively, while compounds **6a** and **8b** have good activity with IC_50_ = 4.66 and 2.16 µM, respectively, compared with the reference drug sorafenib (IC_50_ = 1.28 µM). Molecular docking studies were conducted for compounds **6b**, **7**, and **8b** into putative binding sites of EGFR kinase enzyme, which showed similar binding modes to erlotinib (EGFR kinase inhibitor).

## Supplementary Material

IENZ_1434519_Supplementary_Material.pdf

## References

[1] Eckhardt S. Recent progress in the development of anticancer agents. Curr Med Chem Anticancer Agents 2002;2:419–39. 12678741 10.2174/1568011024606389

[2] Avendańo C, Menéndez J. Medicinal chemistry of anticancer agents. Amsterdam: Elsevier; 2008.

[3] Varmus H. The new era in cancer research. Science 2006;312:1162–5. 16728627 10.1126/science.1126758

[4] Alanazi AM, Alaa A-M, Al-Suwaidan IA, et al Design, synthesis and biological evaluation of some novel substituted quinazolines as antitumor agents. Eur J Med Chem 2014;79:446–54. 24763265 10.1016/j.ejmech.2014.04.029

[5] Al-Suwaidan IA, Abdel-Aziz AA-M, Shawer TZ, et al Synthesis, antitumor activity and molecular docking study of some novel 3-benzyl-4 (3H) quinazolinone analogues. J Enzyme Inhib Med Chem 2016;31:78–89. 10.3109/14756366.2015.1004059 25815668

[6] Mohamed MA, Ayyad RR, Shawer TZ, et al Synthesis and antitumor evaluation of trimethoxyanilides based on 4 (3H)-quinazolinone scaffolds. Eur J Med Chem 2016;112:106–13. 26890117 10.1016/j.ejmech.2016.02.002

[7] Alanazi AM, Al-Suwaidan IA, Alaa A-M, et al Design, synthesis and biological evaluation of some novel substituted 2-mercapto-3-phenethylquinazolines as antitumor agents. Med Chem Res 2013;22:5566–77.

[8] Abdel-Aziz AA, El-Azab AS, Alanazi AM, et al Synthesis and potential antitumor activity of 7-(4-substituted piperazin-1-yl)-4-oxoquinolines based on ciprofloxacin and norfloxacin scaffolds: in silico studies. J Enzyme Inhib Med Chem 2016;31:796–809. 26226179 10.3109/14756366.2015.1069288

[9] Al-Suwaidan IA, Alanazi AM, Abdel-Aziz AA, et al Design, synthesis and biological evaluation of 2-mercapto-3-phenethylquinazoline bearing anilide fragments as potential antitumor agents: molecular docking study. Bioorg Med Chem Lett 2013;23:3935–41. 23683592 10.1016/j.bmcl.2013.04.056

[10] Alanazi AM, Abdel-Aziz AA, Shawer TZ, et al Synthesis, antitumor and antimicrobial activity of some new 6-methyl-3-phenyl-4(3H)-quinazolinone analogues: in silico studies. J Enzyme Inhib Med Chem 2016;31:721–35. 26162029 10.3109/14756366.2015.1060482

[11] Abdel-Aziz AA, El-Azab AS, El-Subbagh HI, et al Design, synthesis, single-crystal and preliminary antitumor activity of novel arenesulfonylimidazolidin-2-ones. Bioorg Med Chem Lett 2012;22:2008–14. 22318157 10.1016/j.bmcl.2012.01.036

[12] El-Azab AS, Al-Omar MA, Alaa A-M, et al Design, synthesis and biological evaluation of novel quinazoline derivatives as potential antitumor agents: molecular docking study. Eur J Med Chem 2010;45:4188–98. 20599299 10.1016/j.ejmech.2010.06.013

[13] Djordjevic VB. Free radicals in cell biology. Int Rev Cytol 2004;237:57–89. 15380666 10.1016/S0074-7696(04)37002-6

[14] Dinkova-Kostova AT, Talalay P. Direct and indirect antioxidant properties of inducers of cytoprotective proteins. Mol Nutr Food Res 2008;52:S128–S38. 18327872 10.1002/mnfr.200700195

[15] Birben E, Sahiner UM, Sackesen C, et al Oxidative stress and antioxidant defense. World Allergy Organ J 2012;5:9–19. 23268465 10.1097/WOX.0b013e3182439613 PMC3488923

[16] Ames BN, Shigenaga MK, Hagen TM. Oxidants, antioxidants, and the degenerative diseases of aging. Proc Natl Acad Sci USA 1993;90:7915–22. 8367443 10.1073/pnas.90.17.7915 PMC47258

[17] Mut-Salud N, Alvarez PJ, Garrido JM, et al Antioxidant intake and antitumor therapy: toward nutritional recommendations for optimal results. Oxid Med Cell Longev 2016;2016:6719534. 26682013 10.1155/2016/6719534 PMC4670692

[18] Joseph A, Shah CS, Kumar SS, et al Synthesis, in vitro anticancer and antioxidant activity of thiadiazole substituted thiazolidin-4-ones. Acta Pharm 2013;63:397–408. 24152899 10.2478/acph-2013-0028

[19] Danciu C, Vlaia L, Fetea F, et al Evaluation of phenolic profile, antioxidant and anticancer potential of two main representants of Zingiberaceae family against B164A5 murine melanoma cells. Biol Res 2015;48:1. 25654588 10.1186/0717-6287-48-1 PMC4417255

[20] Khaledi H, Alhadi AA, Yehye WA, et al Antioxidant, cytotoxic activities, and structure-activity relationship of gallic acid-based indole derivatives. Arch Pharm (Weinheim) 2011;344:703–9. 21953995 10.1002/ardp.201000223

[21] Kodisundaram P, Duraikannu A, Balasankar T, et al Cytotoxic and antioxidant activity of a set of hetero bicylic methylthiadiazole hydrazones: a structure-activity study. Int J Mol Cell Med 2015;4:128–37. 26261802 PMC4499575

[22] Bayomi SM, El-Kashef HA, El-Ashmawy MB, et al Synthesis and biological evaluation of new curcumin analogues as antioxidant and antitumor agents: molecular modeling study. Eur J Med Chem 2015;101:584–94. 26197162 10.1016/j.ejmech.2015.07.014

[23] Manojkumar P, Ravi TK, Subbuchettiar G. Synthesis of coumarin heterocyclic derivatives with antioxidant activity and in vitro cytotoxic activity against tumour cells. Acta Pharm 2009;59:159–70. 19564141 10.2478/v10007-009-0018-7

[24] Bazley LA, Gullick WJ. The epidermal growth factor receptor family. Endocr Relat Cancer 2005;12(Suppl 1):S17–S27. 16113093 10.1677/erc.1.01032

[25] Bishayee S. Role of conformational alteration in the epidermal growth factor receptor (EGFR) function. Biochem Pharmacol 2000;60:1217–23. 11007960 10.1016/s0006-2952(00)00425-1

[26] Hirsch FR, Varella-Garcia M, Bunn PA Jr. et al Epidermal growth factor receptor in non-small-cell lung carcinomas: correlation between gene copy number and protein expression and impact on prognosis. J Clin Oncol 2003;21:3798–807. 12953099 10.1200/JCO.2003.11.069

[27] Ogiso H, Ishitani R, Nureki O, et al Crystal structure of the complex of human epidermal growth factor and receptor extracellular domains. Cell 2002;110:775–87. 12297050 10.1016/s0092-8674(02)00963-7

[28] Ivankovic M, Cukusic A, Gotic I, et al Telomerase activity in HeLa cervical carcinoma cell line proliferation. Biogerontology 2007;8:163–72. 16955216 10.1007/s10522-006-9043-9

[29] Zhang X, Mar V, Zhou W, et al Telomere shortening and apoptosis in telomerase-inhibited human tumor cells. Genes Dev 1999;13:2388–99. 10500096 10.1101/gad.13.18.2388 PMC317024

[30] Wei G, Cui S, Luan W, et al Natural product-based design, synthesis and biological evaluation of Albiziabioside A derivatives that selectively induce HCT116 cell death. Eur J Med Chem 2016;113:92–101. 26922223 10.1016/j.ejmech.2015.12.034

[31] Umekita Y, Ohi Y, Sagara Y, Yoshida H. Co-expression of epidermal growth factor receptor and transforming growth factor-alpha predicts worse prognosis in breast-cancer patients. Int J Cancer 2000;89:484–7. 11102891 10.1002/1097-0215(20001120)89:6<484::aid-ijc3>3.0.co;2-s

[32] Ganjoo KN, Wakelee H. Review of erlotinib in the treatment of advanced non-small cell lung cancer. Biologics 2007;1:335–46. 19707304 PMC2721286

[33] Stamos J, Sliwkowski MX, Eigenbrot C. Structure of the epidermal growth factor receptor kinase domain alone and in complex with a 4-anilinoquinazoline inhibitor. J Biol Chem 2002;277:46265–72. 12196540 10.1074/jbc.M207135200

[34] Minami Y, Shimamura T, Shah K, et al The major lung cancer-derived mutants of ERBB2 are oncogenic and are associated with sensitivity to the irreversible EGFR/ERBB2 inhibitor HKI-272. Oncogene 2007;26:5023–7. 17311002 10.1038/sj.onc.1210292

[35] Rabindran SK, Discafani CM, Rosfjord EC, et al Antitumor activity of HKI-272, an orally active, irreversible inhibitor of the HER-2 tyrosine kinase. Cancer Res 2004;64:3958–65. 15173008 10.1158/0008-5472.CAN-03-2868

[36] Wissner A, Mansour TS. The development of HKI-272 and related compounds for the treatment of cancer. Arch Pharm (Weinheim) 2008;341:465–77. 18493974 10.1002/ardp.200800009

[37] Morgillo F, Martinelli E, Troiani T, et al Antitumor activity of sorafenib in human cancer cell lines with acquired resistance to EGFR and VEGFR tyrosine kinase inhibitors. PLoS One 2011;6:e28841. 22174910 10.1371/journal.pone.0028841 PMC3235154

[38] Yamaguchi N, Lucena-Araujo AR, Nakayama S, et al Dual ALK and EGFR inhibition targets a mechanism of acquired resistance to the tyrosine kinase inhibitor crizotinib in ALK rearranged lung cancer. Lung Cancer 2014;83:37–43. 24199682 10.1016/j.lungcan.2013.09.019 PMC3947244

[39] Yasuda H, de Figueiredo-Pontes LL, Kobayashi S, Costa DB. Preclinical rationale for use of the clinically available multitargeted tyrosine kinase inhibitor crizotinib in ROS1-translocated lung cancer. J Thorac Oncol 2012;7:1086–90. 22617245 10.1097/JTO.0b013e3182570919 PMC3378824

[40] Ou SH. Crizotinib: a novel and first-in-class multitargeted tyrosine kinase inhibitor for the treatment of anaplastic lymphoma kinase rearranged non-small cell lung cancer and beyond. Drug Des Devel Ther 2011;5:471–85. 10.2147/DDDT.S19045 PMC3232174 22162641

[41] Youssef KM, El-Sherbeny MA, El-Shafie FS, et al Synthesis of curcumin analogues as potential antioxidant, cancer chemopreventive agents. Arch Pharm (Weinheim) 2004;337:42–54. 14760627 10.1002/ardp.200300763

[42] Sokmen M, Akram Khan M. The antioxidant activity of some curcuminoids and chalcones. Inflammopharmacology 2016;24:81–6. 27188988 10.1007/s10787-016-0264-5 PMC4883448

[43] Bayomi SM, El-Kashef HA, El-Ashmawy MB, et al Synthesis and biological evaluation of new curcumin derivatives as antioxidant and antitumor agents. Med Chem Res 2013;22:1147–62.

[44] Katsori AM, Hadjipavlou-Litina D. Chalcones in cancer: understanding their role in terms of QSAR. Curr Med Chem 2009;16:1062–81. 19275612 10.2174/092986709787581798

[45] Karthikeyan C, Moorthy NS, Ramasamy S, et al Advances in chalcones with anticancer activities. Recent Pat Anticancer Drug Discov 2015;10:97–115. 25138130 10.2174/1574892809666140819153902

[46] Syam S, Abdelwahab SI, Al-Mamary MA, Mohan S. Synthesis of chalcones with anticancer activities. Molecules 2012;17:6179–95. 22634834 10.3390/molecules17066179 PMC6268294

[47] Jung SK, Lee MH, Lim DY, et al Isoliquiritigenin induces apoptosis and inhibits xenograft tumor growth of human lung cancer cells by targeting both wild type and L858R/T790M mutant EGFR. J Biol Chem 2014;289:35839–48. 25368326 10.1074/jbc.M114.585513 PMC4276852

[48] Xu YY, Cao Y, Ma H, et al Design, synthesis and molecular docking of alpha,beta-unsaturated cyclohexanone analogous of curcumin as potent EGFR inhibitors with antiproliferative activity. Bioorg Med Chem 2013;21:388–94. 23245570 10.1016/j.bmc.2012.11.031

[49] Alswah M, Bayoumi AH, Elgamal K, et al Design, synthesis and cytotoxic evaluation of novel chalcone derivatives bearing triazolo[4,3-a]-quinoxaline moieties as potent anticancer agents with dual EGFR kinase and tubulin polymerization inhibitory effects. Molecules 2017;23:E48 DOI:10.3390/molecules23010048 29280968 PMC5943945

[50] Lee JY, Lee YM, Chang GC, et al Curcumin induces EGFR degradation in lung adenocarcinoma and modulates p38 activation in intestine: the versatile adjuvant for gefitinib therapy. PLoS One 2011;6:e23756. 21858220 10.1371/journal.pone.0023756 PMC3157465

[51] Wada K, Lee JY, Hung HY, et al Novel curcumin analogs to overcome EGFR-TKI lung adenocarcinoma drug resistance and reduce EGFR-TKI-induced GI adverse effects. Bioorg Med Chem 2015;23:1507–14. 25753330 10.1016/j.bmc.2015.02.003 PMC4782611

[52] Starok M, Preira P, Vayssade M, et al EGFR inhibition by curcumin in cancer cells: a dual mode of action. Biomacromolecules 2015;16:1634–42. 25893361 10.1021/acs.biomac.5b00229

[53] Qin HL, Leng J, Youssif BGM, et al Synthesis and mechanistic studies of curcumin analog-based oximes as potential anticancer agents. Chem Biol Drug Des 2017;90:443–9. 28186369 10.1111/cbdd.12964

[54] Li QS, Lv XH, Zhang YB, et al Identification of novel 3,5-diarylpyrazoline derivatives containing salicylamide moiety as potential anti-melanoma agents. Bioorg Med Chem Lett 2012;22:6596–601. 23025996 10.1016/j.bmcl.2012.09.004

[55] Liu JJ, Zhang H, Sun J, et al Synthesis, biological evaluation of novel 4,5-dihydro-2H-pyrazole 2-hydroxyphenyl derivatives as BRAF inhibitors. Bioorg Med Chem 2012;20:6089–96. 22985957 10.1016/j.bmc.2012.08.020

[56] Kaur N, Kishore D. Application of chalcones in heterocycles synthesis: synthesis of 2-(isoxazolo, pyrazolo and pyrimido) substituted analogues of 1, 4-benzodiazepin-5-carboxamides linked through an oxyphenyl bridge. J Chem Sci 2013;125:555–60.

[57] Samshuddin S, Narayana B, Sarojini B, et al Synthesis, characterization and biological evaluation of functionalized derivatives of versatile synthon 4, 4'-difluoro chalcone. Der Pharma Chemica 2012;4:1445–7.

[58] Bingul M, Tan O, Gardner CR, et al Synthesis, characterization and anti-cancer activity of hydrazide derivatives incorporating a quinoline moiety. Molecules 2016;21:916 DOI:10.3390/molecules21070916 PMC6273134 27428941

[59] Sangani CB, Makawana JA, Zhang X, et al Design, synthesis and molecular modeling of pyrazole-quinoline-pyridine hybrids as a new class of antimicrobial and anticancer agents. Eur J Med Chem 2014;76:549–57. 24607998 10.1016/j.ejmech.2014.01.018

[60] Lv PC, Li DD, Li QS, et al Synthesis, molecular docking and evaluation of thiazolyl-pyrazoline derivatives as EGFR TK inhibitors and potential anticancer agents. Bioorg Med Chem Lett 2011;21:5374–7. 21802290 10.1016/j.bmcl.2011.07.010

[61] Demnitz J, LaVecchia L, Bacher E, et al Enantiodivergent synthesis of (R)-and (S)-rolipram. Molecules 1998;3:107–19.

[62] Mosmann T. Rapid colorimetric assay for cellular growth and survival: application to proliferation and cytotoxicity assays. J Immunol Methods 1983;65:55–63. 6606682 10.1016/0022-1759(83)90303-4

[63] Denizot F, Lang R. Rapid colorimetric assay for cell growth and survival. Modifications to the tetrazolium dye procedure giving improved sensitivity and reliability. J Immunol Methods 1986;89:271–7. 3486233 10.1016/0022-1759(86)90368-6

[64] Hsu CF, Peng H, Basle C, et al ABTS•+ scavenging activity of polypyrrole, polyaniline and poly (3, 4‐ethylenedioxythiophene). Polym Int 2011;60:69–77.

[65] Lissi EA, Modak B, Torres R, et al Total antioxidant potential of resinous exudates from Heliotropium species, and a comparison of the ABTS and DPPH methods. Free Radic Res 1999;30:471–7. 10400459 10.1080/10715769900300511

[66] Available from: http://www.abcam.com/human-egfr-in-cell-elisa-kit-ab126419.html [last accessed 15 Dec 2017].

[67] MOE 200810 of Chemical Computing Group Inc. Available from: http://www.chemcomp.com/ [last accessed 1 Jun 2017].

[68] El-Azab AS, Abdel-Aziz AA, Ghabbour HA, Al-Gendy MA. Synthesis, in vitro antitumour activity, and molecular docking study of novel 2-substituted mercapto-3-(3,4,5-trimethoxybenzyl)-4(3H)-quinazolinone analogues. J Enzyme Inhib Med Chem 2017;32:1229–39. 28948843 10.1080/14756366.2017.1368504 PMC6010141

[69] El-Azab AS, Al-Dhfyan A, Abdel-Aziz AA, et al Synthesis, anticancer and apoptosis-inducing activities of quinazoline-isatin conjugates: epidermal growth factor receptor-tyrosine kinase assay and molecular docking studies. J Enzyme Inhib Med Chem 2017;32:935–44. 28718672 10.1080/14756366.2017.1344981 PMC6445199

[70] El-Sayed MA, El-Husseiny WM, Abdel-Aziz NI, et al Synthesis and biological evaluation of 2-styrylquinolines as antitumour agents and EGFR kinase inhibitors: molecular docking study. J Enzyme Inhib Med Chem 2018;33:199–209. 29251017 10.1080/14756366.2017.1407926 PMC7012010

[71] Lv Q, Fang L, Wang P, et al A simple one-pot synthesis of quinoline-4-carboxylic acid derivatives by Pfitzinger reaction of isatin with ketones in water. Monatshefte Chemie-Chemical Monthly 2013;144:391–4.

[72] Vega-Avila E, Pugsley MK. An overview of colorimetric assay methods used to assess survival or proliferation of mammalian cells. Proc West Pharmacol Soc 2011;54:10–14. 22423572

[73] Available from: http://www.sigmaplot.co.uk/products/sigmaplot/sigmaplot-details.php [last accessed 1 Jul 2017].

