# Supplementary material for: Synthesis, antitumour and antioxidant activities of novel α,β-unsaturated ketones and related heterocyclic analogues: EGFR inhibition and molecular modelling study
Source: J Enzyme Inhib Med Chem. 2018 Feb 19;33(1):507–18. doi: 10.1080/14756366.2018.1434519 (PMC6010098; doi:10.1080/14756366.2018.1434519)
Supplement: IENZ_1434519_Supplementary_Material.pdf [file IENZ_A_1434519_SM4294.pdf]

Researcher : Dr/Magda Abdelaziz email: Magdaaziz1@yahoo.com *mob.01006396439*
Assay : EGFR enzyme assay
Samples : 4 preparations.
Date : 18-12-2017
Reference : **Sorafinib**
Cell line : ---
Reader : ROBONIK P2000 ELISA READER wl 450 nm
Kit used for immune-assay: *cloud clone* ***SEA757Hu 96 Tests***
Solvent : DMSO
Assay samples :Cell culture supernatants

EGFR report

ser	compound	M.W	IC50	EGFR IC50 uM
**1**	**6a**	**285.15**	9.4±0.98	4.66
**2**	**6b**	**299.20**	6.7±0.67	0.56
**3**	**7**	**368.25**	10.3±1.13	1.6
**4**	**8b**	**397.00**	5.5±0.45	2.16
5	**Sorafinib**	393.44	0.74±0.03	1.28

*Ref=**Sorafinib**

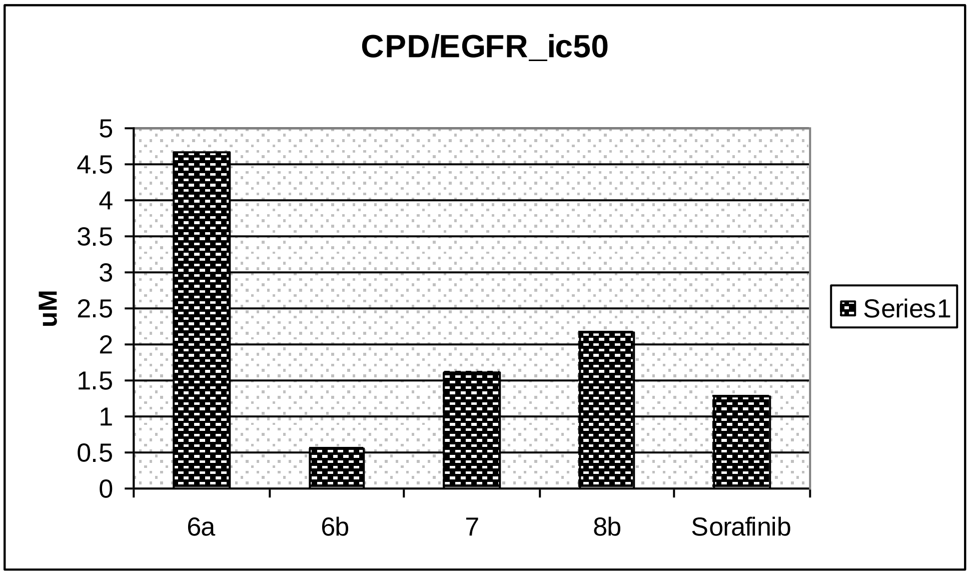

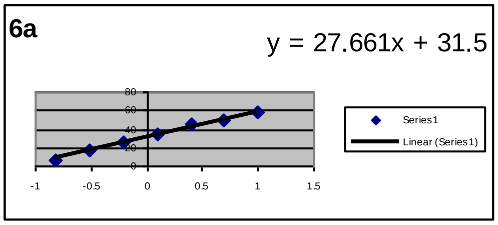
6a
y = 27.661x + 31.5
80
60
40
20
0
-1
-0.5
0
0.5
1
1.5
Series1
Linear (Series1)

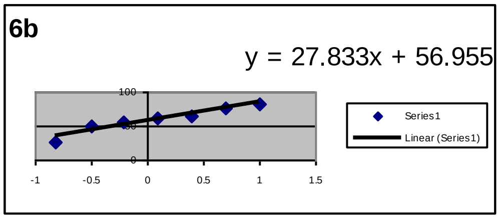
6b
y = 27.833x + 56.955
100
50
0
-1
-0.5
0
0.5
1
1.5
Series1
Linear (Series1)

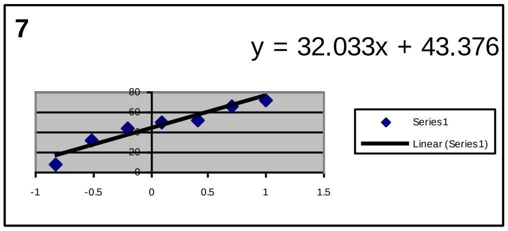
7
y = 32.033x + 43.376
80
60
40
20
0
-1
-0.5
0
0.5
1
1.5
Series1
Linear (Series1)

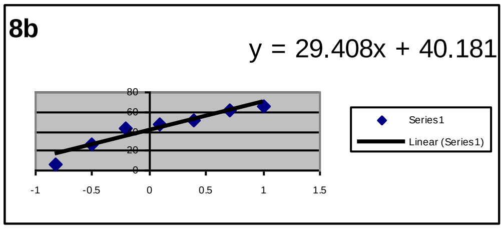
8b
y = 29.408x + 40.181
80
60
40
20
0
-1
-0.5
0
0.5
1
1.5
Series1
Linear (Series1)

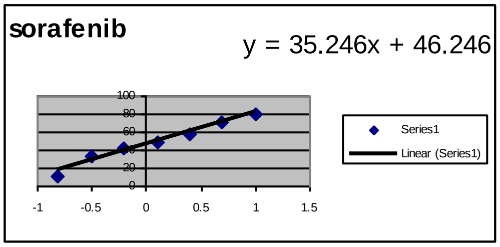
sorafenib
y = 35.246x + 46.246
100
80
60
40
20
0
-1
-0.5
0
0.5
1
1.5
Series1
Linear (Series1)

Detailed Results:

ST.	CONC.ng/ml
St.1	200
St.2	100
St.3	50
St.4	25
St.5	12.5
St.6	6.25
St.7	3.12

plate map:

	1	2	3	4	5	6
A	St.1	**6a**(10uM)	**6b**(10uM)	**7**(10uM)	**8b**(10uM)	**ref**(10uM)
B	St.2	**6a**(5uM)	**6b**(5uM)	**7**(5uM)	**8b**(5uM)	**ref**(5uM)
C	St.3	**6a**(2.5uM)	**6b**(2.5uM)	**7**(2.5uM)	**8b**(2.5uM)	**ref**(2.5uM)
D	St.4	**6a**(1.25uM)	**6b**(1.25uM)	**7**(1.25uM)	**8b**(1.25uM)	**ref**(1.25uM)
E	St.5	**6a**(0.62uM)	**6b**(0.62uM)	**7**(0.62uM)	**8b**(0.62uM)	**ref**(0.62uM)
F	St.6	**6a**(0.31uM)	**6b**(0.31uM)	**7**(0.31uM)	**8b**(0.31uM)	**ref**(0.31uM)
G	St.7	**6a**(0.15uM)	**6b**(0.15uM)	**7**(0.15uM)	**8b**(0.15uM)	**ref**(0.15uM)
H	B	cont	cont	cont	cont	cont

Samples OD results

*I

	1	2	3	4	5	6
A	2.746	0.708	0.257	**0.438**	0.578	**0.296**
B	1.133	0.836	0.372	**0.556**	0.646	0.463
C	0.728	0.939	0.588	**0.784**	0.826	0.715
D	0.376	1.115	0.637	**0.843**	0.881	0.841
E	0.115	1.261	0.741	0.955	0.937	0.957
F	0.044	1.417	0.861	1.173	1.264	1.125
G	0.019	1.639	1.264	1.603	1.629	1.556
H	0.005	0.008	0.003	1.771	1.759	1.779

**II

	7	8	9	10	11	12
A	2.749	0.697	0.264	**0.443**	0.574	**0.315**
B	1.141	0.829	0.377	**0.558**	0.651	0.457
C	0.735	0.942	0.579	**0.791**	0.833	0.723
D	0.384	1.124	0.642	**0.847**	0.894	0.855
E	0.122	1.263	0.746	0.965	0.962	0.963
F	0.048	1.433	0.854	1.168	1.282	1.137
G	0.016	1.645	1.272	1.621	1.644	1.569
H	0.011	0.005	0.007	1.759	1.766	1.781

2.746	2.749	0.708	0.697	0.257	0.264	**0.438**	**0.443**	0.578	0.574	**0.296**	**0.315**
1.133	1.141	0.836	0.829	0.372	0.377	**0.556**	**0.558**	0.646	0.651	0.463	0.457
0.728	0.735	0.939	0.942	0.588	0.579	**0.784**	**0.791**	0.826	0.833	0.715	0.723
0.376	0.384	1.115	1.124	0.637	0.642	**0.843**	**0.847**	0.881	0.894	0.841	0.855
0.115	0.122	1.261	1.263	0.741	0.746	0.955	0.965	0.937	0.962	0.957	0.963
0.044	0.048	1.417	1.433	0.861	0.854	1.173	1.168	1.264	1.282	1.125	1.137
0.019	0.016	1.639	1.645	1.264	1.272	1.603	1.621	1.629	1.644	1.556	1.569
0.005	0.011	0.008	0.005	0.003	0.007	1.771	1.759	1.759	1.766	1.779	1.781

s	conc uM	log conc	%inh	ic50
	10	1	57.65011	
	5	0.69897	50.34841	
	2.5	0.39794	44.35137	
	1.25	0.09691	34.55152	
**6a**	0.625	-0.20761	26.84952	4.66
	0.31	-0.50864	18.1616	
	0.15	-0.82391	6.671609	
	10	1	83.66197	
	5	0.69897	76.71609	
	2.5	0.39794	64.44774	
	1.25	0.09691	61.23795	
**6b**	0.625	-0.20761	55.33729	0.56
	0.31	-0.50864	48.95478	
	0.15	-0.82391	26.52335	
	10	1	72.78725	
	5	0.69897	65.98221	
	2.5	0.39794	52.86138	
	1.25	0.09691	49.65159	
**7**	0.625	-0.20761	43.2765	1.6
	0.31	-0.50864	31.78651	
	0.15	-0.82391	8.228317	
	10	1	64.8851	
	5	0.69897	60.72646	
**8b**	2.5	0.39794	50.51149	2.16
	1.25	0.09691	47.28688	

	0.625	-0.20761	43.85471	
	0.31	-0.50864	26.25649	
	0.15	-0.82391	6.968125	
**Sorafinib**	10	1	80.88955	
	10	0.69897	71.63825	
	5	0.39794	56.7235	
	2.5	0.09691	49.4811	
	1.25	-0.20761	43.2765	1.28
	0.625	-0.50864	33.92884	
	0.31	-0.82391	10.82283	

Sample	Wells	Raw	Background Corrected	Conc.	Conc. (Average)	%CV	SD	SEM
6a(10uM)	A3	0.708	0.696	57.55	57.13	1.04	0.594	0.42
	A4	0.697		56.7				
6a(5uM)	B3	0.836	0.826	67.25	66.98	0.556	0.373	0.263
	B4	0.829		66.72				
6a(2.5uM)	C3	0.939	0.934	74.96	75.07	0.21	0.158	0.112
	C4	0.942		75.18				
6a(1.25uM)	D3	1.12	1.11	87.96	88.29	0.528	0.467	0.33
	D4	1.12		88.62				
6a(0.62uM)	E3	1.26	1.26	98.61	98.68	0.104	0.103	0.0725
	E4	1.26		98.75				
6a(0.31uM)	F3	1.42	1.42	109.9	110.4	0.735	0.812	0.574
	F4	1.43		111				
6a(0.15uM)	G3	1.64	1.64	125.7	125.9	0.239	0.301	0.213
	G4	1.65		126.1				
6b(10uM)	A5	0.257	0.254	21.75	22.04	1.88	0.414	0.293
	A6	0.264		22.33				
6b(5uM)	B5	0.372	0.368	31.21	31.41	0.911	0.286	0.202
	B6	0.377		31.61				
6b(2.5uM)	C5	0.588	0.577	48.31	47.96	1.03	0.495	0.35
	C6	0.579		47.61				
6b(1.25uM)	D5	0.637	0.633	52.1	52.29	0.521	0.272	0.193
	D6	0.642		52.48				
6b(0.62uM)	E5	0.741	0.737	60.06	60.25	0.446	0.269	0.19
	E6	0.746		60.44				
6b(0.31uM)	F5	0.861	0.851	69.12	68.86	0.54	0.372	0.263
	F6	0.854		68.6				

6b(0.15uM)	G5<br>G6	1.26<br>1.27	1.26	98.83<br>99.41	99.12	0.414	0.41	0.29
7(10uM)	A7<br>A8	0.438<br>0.443	0.434	36.51<br>36.91	36.71	0.768	0.282	0.199
7(5uM)	B7<br>B8	0.556<br>0.558	0.551	45.82<br>45.97	45.89	0.24	0.11	0.078
7(2.5uM)	C7<br>C8	0.784<br>0.791	0.781	63.32<br>63.85	63.59	0.589	0.374	0.265
7(1.25uM)	D7<br>D8	0.843<br>0.847	0.839	67.77<br>68.07	67.92	0.313	0.213	0.15
7(0.62uM)	E7<br>E8	0.955<br>0.965	0.954	76.15<br>76.89	76.52	0.687	0.526	0.372
7(0.31uM)	F7<br>F8	1.17<br>1.17	1.16	92.2<br>91.84	92.02	0.281	0.258	0.183
7(0.15uM)	G7<br>G8	1.6<br>1.62	1.61	123.2<br>124.4	123.8	0.73	0.904	0.639
8b(10uM)	A9<br>A10	0.578<br>0.574	0.57	47.53<br>47.22	47.37	0.464	0.22	0.156
8b(5uM)	B9<br>B10	0.646<br>0.651	0.642	52.79<br>53.18	52.98	0.514	0.272	0.192
8b(2.5uM)	C9<br>C10	0.826<br>0.833	0.823	66.49<br>67.02	66.76	0.558	0.373	0.264
8b(1.25uM)	D9<br>D10	0.881<br>0.894	0.881	70.62<br>71.6	71.11	0.967	0.688	0.486
8b(0.62uM)	E9<br>E10	0.937<br>0.962	0.943	74.81<br>76.67	75.74	1.74	1.32	0.93
8b(0.31uM)	F9<br>F10	1.26<br>1.28	1.27	98.83<br>100.1	99.48	0.927	0.923	0.652
8b(0.15uM)	G9<br>G10	1.63<br>1.64	1.63	125<br>126.1	125.5	0.599	0.752	0.532
ref(10uM)	A11<br>A12	0.296<br>0.315	0.299	24.99<br>26.56	25.78	4.3	1.11	0.783
ref(5uM)	B11<br>B12	0.463<br>0.457	0.454	38.5<br>38.02	38.26	0.88	0.337	0.238
ref(2.5uM)	C11<br>C12	0.715<br>0.723	0.713	58.08<br>58.69	58.38	0.739	0.431	0.305
ref(1.25uM)	D11<br>D12	0.841<br>0.855	0.842	67.62<br>68.67	68.15	1.09	0.744	0.526
ref(0.62uM)	E11<br>E12	0.957<br>0.963	0.954	76.29<br>76.74	76.52	0.412	0.315	0.223
ref(0.31uM)	F11<br>F12	1.13<br>1.14	1.12	88.69<br>89.57	89.13	0.697	0.622	0.44

ref(0.15uM)	G11	1.56	1.56	119.8	120.3	0.544	0.654	0.463
	G12	1.57		120.7				
cont	H7	1.77	1.76	135	134.9	0.499	0.673	0.275
	H8	1.76		134.2				
	H9	1.76		134.2				
	H10	1.77		134.7				
	H11	1.78		135.6				
	H12	1.78		135.7				
Blank	H1	0.005	0	< Curve	-	-	-	-
	H2	0.011		< Curve				
	H3	0.008		< Curve				
	H4	0.005		< Curve				
	H5	0.003		< Curve				
	H6	0.007		< Curve				
